# Tumoral and circulating genomic landscape inform survival differences in colorectal carcinomatosis

**DOI:** 10.1016/j.tranon.2025.102379

**Published:** 2025-04-03

**Authors:** Michael G. White, Reed I. Ayabe, Mohammad A. Zeineddine, Fadl A. Zeineddine, Abdelrahman M.G. Yousef, Mahmoud Yousef, Norman J. Galbraith, J․Bryan Iorgulescu, Christopher Scally, Keith Fournier, Timothy E. Newhook, Nancy Y. You, Jason Willis, Scott Kopetz, George J. Chang, John Paul Shen, Abhineet Uppal

**Affiliations:** a Department of Colon and Rectal Surgery, The University of Texas MD Anderson Cancer Center, Houston, TX, USA; b Department of Surgical Oncology, The University of Texas MD Anderson Cancer Center, Houston, TX, USA; c Department of Surgery, University of California Irvine Medical Center, Orange, CA, USA; d Department of Gastrointestinal Medical Oncology, The University of Texas MD Anderson Cancer Center, Houston, TX, USA; e Now at Department of Surgery, Emory University School of Medicine, Atlanta, GA, USA

**Keywords:** Colorectal cancer, Circulating tumoral DNA, Peritoneal metastasis, Liver metastasis, BRAF mutation, KRAS mutation

## Abstract

Colorectal peritoneal metastases (CPM) are the third most common site of metastatic spread of colorectal cancer and are associated with worse survival than other sites of metastatic disease. In recent years tumoral circulating tumoral DNA (ctDNA) mutational status has been increasingly utilized in clinical decision making for metastatic colorectal cancer patients despite its utility in CPM being poorly understood. Here we describe standard of care performed mutational profiles and associated outcomes for unresectable CPM patients, with a contextual comparison to 160 unresected colorectal liver metastases (CLM) patients. Of 508 patients, 288 (57 %) had CPM alone and 220 (43 %) had CPM with extraperitoneal metastases. Patients with synchronous CPM and CLM had worse overall survival (HR 1.67 [95 %CI 1.26–2.22]). Mutations in ctDNA were noted in 110/145 (75.9 %) of CPM patients, with mutations in *KRAS* or *PIK3CA* ctDNA being associated with worse survival. Importantly, the association between tumoral mutational profile and survival differed by site of metastatic disease. The prognostic significance of specific mutations, particularly *BRAF* and *KRAS*, differs between patients with CPM and CLM, and supports the distinct biology of these metastatic sites and the importance of tissue and circulating genomic profiling to risk-stratify these patients according to site of metastasis.

## Highlights

• The mutational profile and prognostic value of CPM are distinct from CLM.
• Rates of ctDNA positivity were lower in patients with isolated CPM.
• Improved survival was noted in CPM patients treated with FOLFIRI and Bevacizumab.

## Introduction

The peritoneum is the third most common site of metastasis for colorectal cancer (CRC) and is a particularly challenging site of secondary disease for both medical and surgical oncologists with worse survival for patients when compared to liver or lung metastases. Historically, cancer registry data has demonstrated a median overall survival (OS) of only 13 months for patients with colorectal peritoneal metastases (CPM), compared to 22 months for patients with unresectable colorectal liver metastases (CLM) [1,2]. The increasing utilization of cytoreductive surgery for patients with CPM has provided select patients with prolonged and even long-term survival. Patients with CPM who are candidates for cytoreductive surgery (CRS) have a reported median OS of 42 months in the recent PRODIGE 7 trial [3]. However, most patients presenting with CPM are not cytoreduction candidates. Although not specifically studied in patients with CPM, reports of contemporary multi-drug chemotherapy regimens (including anti-*EGFR*, anti-VEGF antibodies and combined oxaliplatin and irinotecan) also appear to offer improved survival to patients with unresectable CRC [4]. The outcomes of CPM patients treated with contemporary multi-drug chemotherapy regimens, and the prognostic and predictive association with specific driver mutations, remain largely undefined by prior studies that were limited by lack of mutation status, detailed pathology data, and detailed systemic therapy data.

Clinical decision making in CRC for contemporary patients is aided both by the use of tissue and circulating tumoral DNA (ctDNA) mutational profiling of patient tumors, typically in the case of locally advanced or metastatic disease. Prior retrospective studies of CPM cohorts have shown that *BRAF* p.V600E mutations are associated with worse survival, while detrimental survival effects of *KRAS* mutations were not consistently observed[5,6]. Recognizing that CPM represents a distinct subset of CRC disease biology, it is notable that comprehensive mutational profiling has not been performed for these tumors outside of small cohorts. This represents a significant gap in knowledge for both medical and surgical oncologists [7,8].

Here we describe the outcomes of patients with CPM treated at a single center with contemporary multidrug chemotherapeutic regimens, along with analysis of standard of care tumoral and ctDNA mutational profiling. Patient outcomes were correlated to the mutational status of tumors and also compared to ctDNA profiles. For context, we compared these to a cohort of patients with genomically profiled CLM treated in a similar fashion. Given its distinct biologic behavior, we hypothesized that CPM would display a unique genomic profile and molecular indicators of prognosis. This work will aid in identifying optimal markers of disease biology for this unique patient population and inform treatment decisions and prognostication.

## Methods

### Patient identification and data collection

All consecutive patients with CPM or CLM from treated at the University of Texas MD Anderson Cancer Center in Houston, Texas between 2006 and 2020 were retrospectively identified from an IRB approved clinical database of colorectal cancer patients developed with the Foundry platform, which uses computational tools to categorize clinical and laboratory data from multiple sources within the institution's clinical and laboratory records (Palantir Inc, Palo Alto CA) [9]. Patients whose initial metastatic presentation included peritoneal metastases based on radiographic findings or pathology were selected for the CPM cohort. Patients who did not receive treatment at MD Anderson or developed peritoneal disease subsequent to metastases at other sites were excluded. Patients whose initial metastatic presentation included CLM without peritoneal metastases and did not undergo surgical resection were selected for the CLM cohort and used as a comparator against the overall CPM cohort. This study was approved by the Institutional Review Board of MD Anderson Cancer Center.

Patient demographics, primary tumor characteristics, mutational analyses of tumor, analyses of mutated ctDNA levels, systemic therapy regimens and follow-up data were obtained from institutional databases and augmented by primary medical record review. Circulating tumoral DNA was considered positive if a pathogenic or tumor specific mutation was detected above the threshold defined by a specific assay. These patients were grouped as having clinically positive ctDNA and analyzed as such against those without circulating mutations or mutations detected below an assay's defined threshold. All patients were evaluated by a medical oncologist and treated with systemic therapy as appropriate. Clinical data from the electronic health record was used to conduct the analysis after integration into a cloud-based tool within the Context Engine, MD Anderson's data management system.

### Outcomes

Overall survival (OS) after diagnosis of CPM was defined as the time interval from the first imaging, pathologic or clinical identification of peritoneal metastasis to the date of last contact or date of death. Patients were grouped as CPM only or CPM with extra-peritoneal metastases based on imaging or pathologic findings. Secondary outcomes included OS by mutation status of the ten most commonly mutated genes within the cohort, and OS by patient and tumor characteristics (age, gender, race, ethnicity, smoking status, histologic subtype and primary tumor grade).

### Sequencing details

Sequencing was performed at a Clinical Lab Improvement Amendment (CLIA)-certified molecular diagnostics laboratory at MD Anderson Cancer Center. Patients underwent sequencing to identify somatic mutations in genes listed in Table 1. For detection of mutations in the coding sequence of tissue, DNA was extracted from formalin-fixed, paraffin-embedded tumor samples and sequenced on one of two next-generation sequencing (NGS)-based, matched tumor-normal platforms: 1) PCR-amplicon-based target capture of 134 genes using the Ion GeneStudio S5 Prime System with analysis using Torrent Suite and Ion Reporter [10]; or 2) hybridization capture-based target capture of 610 genes using the NovaSeq 6000 platform with analysis using the Illumina Real Time Analysis Software and MDA MAPP BIP v1.0. A minimum sequencing depth of 250 × was considered as adequate for the first panel and 100x for the second panel. A variant allelic frequency of at least 5 % was used as the cutoff for variant calling. *KRAS* mutation subtype was stratified as reported in a recent publication by Tonello and colleagues [11].

**Table 1:** Characteristics of patients with colorectal peritoneal metastases (CPM).

**Characteristic**	**CPM only (*n*=288)**	**CPM + other metastatic site (*n*=220)**	**p value**
**Age at Diagnosis, years, median (IQR)**	55.7 (44.9–63.5)	54.3 (47.7–62.4)	0.601
**Gender, n ( %)**			0.869
Male	163 (56.6)	127 (57.7)	
Female	125 (43.4)	93 (42.3)	
**Race, n ( %)**			0.510
White/Caucasian	201 (69.8)	144 (65.5)	
Black	41 (14.2)	35 (15.9)	
Asian	17 (5.9)	16 (7.3)	
American Native	2 (0.7)	2 (1.0)	
Other/Unknown	27 (9.4)	23 (10.5)	
**Ethnicity, n ( %)**			0.632
Hispanic/Latino	40 (13.9)	31 (14.1)	
Other	235 (81.6)	178 (80.9)	
Unknown	13 (4.5)	11 (5.0)	
**Primary Histologic Grade, n ( %)**			0.001
Well-Differentiated	1 (0.3)	0 (0.0)	
Moderately Differentiated	208 (72.2)	193 (87.7)	
Poorly Differentiated	61 (21.2)	19 (8.6)	
Unknown	18 (6.2)	8 (3.6)	
**Histologic Subtype, n ( %)**			0.001
Mucinous	107 (37.2)	54 (24.5)	
Signet ring cell	29 (10.1)	5 (2.3)	
Adenocarcinoma, NOS	152 (52.8)	161 (73.2)	
**Other Metastases, n ( %)**			
Liver	–	180 (81.8)	
Lung	–	36 (16.4)	
Other	–	4 (1.8)	
**Smoking History, n ( %)**			0.510
Yes	103 (35.8)	72 (32.7)	
No	185 (64.2)	148 (67.3)	

Additionally, tumor agnostic NGS of circulating tumor DNA (ctDNA) for 70 genes was performed on patients’ plasma samples using the NextSeq instrument (Supplemental Table 2). Analysis of ctDNA sequencing data was performed using Illumina Realtime Analysis Software and Bioinformatics Pipeline. The effective lower limit of detection for this assay was validated to be a VAF of 0.3 % at 30 ng of cell-free DNA input or VAF of 1 % at 5 ng of cell-free DNA input [12]. The GRCh37/hg19 reference was used for all sequencing analyses. These assays were not validated for detection of copy number losses.

### Statistical analysis

The mutation frequency for selected genes were compared to prior CLM data and the Tumor Cancer Genome Atlas colon cancer (COAD) dataset [13,14]. Univariable comparisons were performed for categorical variables using Fisher's Exact Test for sparse data. Comparisons between continuous variables were performed using the Kruskal-Wallis test.

For survival analyses, OS after diagnosis of peritoneal metastasis was defined from the time of carcinomatosis diagnosis to death, with censoring at date of last follow-up. Date of diagnosis was determined using clinical and radiographic reports. Last follow-up was determined using the institutional tumor registry and clinical records. OS was estimated using the Kaplan-Meier method and the hazard ratio for survival was determined using the Cox proportional hazards method. Correction for multiple testing of genes’ association with OS was performed using the method of Benjamini and Hochberg, using *m* (*number of tests*) of 10 and *Q* (*false discovery rate*) of 10 % [15]. Based on an overall event rate of 50 % at 3 years, this study has an 80 % power with an alpha of 0.05 to detect a hazard ratio of 1.5 in Cox survival analysis of mutations occurring with at least a 23 % frequency. All statistical analyses were performed using R (Vienna, Austria) within the Palantir Foundry platform (Denver, Colorado).

## Results

### Patient characteristics

A total of 526 patients with CPM treated from January 1, 2006 to December 31, 2020 were identified. The median age of the cohort was 54.3 years [IQR 46.7–62.2] and 227 (43.2 %) were female. Eighteen patients who underwent cytoreductive surgery were excluded, leaving 508 patients for analysis (Supplementary Figure 1) (Table 1). Three hundred patients (59.1 %) presented with synchronous CPM while 208 presented with metachronous CPM, for whom the median disease-free interval between primary tumor resection and peritoneal metastasis diagnoses was 17.9 months (95 % CI: 16.2–19.7). At the time of CPM diagnosis, 36 (7.1) also had lung metastases and 180 (35.4 %) had liver metastases. Patients with CPM alone were more likely to have poorly differentiated histology (21.2 % versus 8.6 %, *p* < 0.001) and signet ring cell carcinoma (SRC) (10.1 % versus 2.3 %, *p* < 0.001), as compared to those with CPM and extra-peritoneal metastases. There were no other significant differences observed in baseline patient demographics or primary tumor characteristics (Table 1). Disease-free interval between primary tumor resection and CPM diagnosis was not associated with primary tumor grade (moderately differentiated: 18.1 (95 % CI: 16.3–20.3) months; poorly differentiated: 14.3 (95 % CI: 10.4- 39.1) months; *p* = 0.45*)*. All patients received multiagent 5-FU-based systemic therapy and most received multiple lines of treatment: 401 (78.9 %) received FOLFOX and 359 (70.7 %) received FOLFIRI. Additionally, 348 (68.5 %) received bevacizumab, 148 (29.1 %) received anti-EGFR therapy, 115 (22.6 %) received Trifluridine/Tipiracil (Lonsurf), and 12 (2.4 %) received BRAF inhibition during their treatment for metastatic disease.

### Survival among colorectal cancer patients with peritoneal metastases

**Fig. 1:**
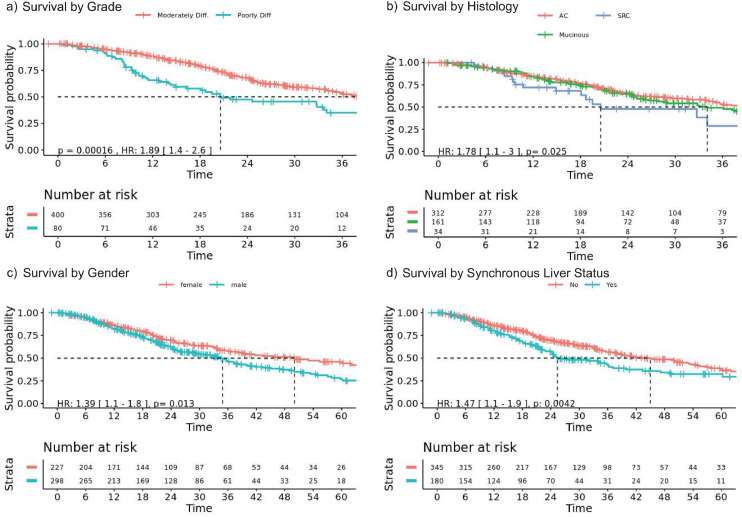
Overall survival after diagnosis of peritoneal metastases. (a) Patients with poorly differentiated primary tumors (blue) had a shorter median overall survival (OS) than those with moderately differentiated primary tumors (20.6 months versus 39.1 months, *p* = 1.2 × 10^–4^). (b) Patients with signet ring cell (SRC) carcinoma had shorter median OS than non-mucinous adenocarcinomas (AC) 20.6 versus 41.8 months, *p* = 0.025). (c) Male patients had shorter median OS than female patients (34.9 versus 50.0 months, *p* = 0.013). (d) Patients with synchronous liver metastases had worse median OS than those with peritoneal metastases alone (25.4 versus 45.0 months, *p* = 0.004).

Median OS for the entire CPM cohort was 37.5 months (95 % CI: 34.1–47.1) after diagnosis with CPM. Comparing moderately and poorly differentiated tumors within the CPM cohort we see that poorly differentiated tumors were associated with a significantly diminished OS on univariable analysis (HR 1.89, 95 % CI: 1.4–2.6, *p* < 0.001, Fig. 1a). While on multivariable analysis (Supplementary Table 1), poorly differentiated tumors (HR 2.08 vs. well-differentiated as reference, 95 % CI:1.42–3.04, *p* < 0.001), identified in 80 (15.7 %) of patients (Fig. 1a), and signet ring cell histology (HR 2.35 vs. conventional histology, 95 % CI:1.41–3.92, *p* = 0.001), identified in 34 (6.7 %) of patients (Fig. 1b).

Age at diagnosis (HR 1.02, 95 % CI: 1.01–1.04, *p* = 0.004) and male sex OS (HR 1.37, 95 % CI: 1.05–1.78, *p* = 0.019) were associated with shorter OS (Supplementary Table 1) (Fig. 1c). Patients with synchronous liver metastases had worse survival than those with peritoneal metastases alone on univariable (Fig. 1d) and multivariable analysis (HR 1.67, 95 % CI: 1.26–2.22, *p* < 0.001) (Supplementary Table 1). However, a difference in OS was not observed for patients with CPM and lung metastases when compared to CPM alone (HR 1.34, 95 % CI: 0.90–1.97, *p* = 0.146).

Analysis of chemotherapy regimen demonstrated that receipt of FOLFIRI (HR 0.69, 95 % CI: 0.52–0.90, *p* = 0.008) and bevacizumab (HR 0.73, 95 % CI 0.56–0.96, *p* = 0.023) were associated with prolonged OS. Receipt of FOLFOX was not associated with any difference in survival (HR 0.81, 95 % CI: 1.23–0.60, *p* = 0.17). Similarly, receipt of anti-EGFR therapy (HR 0.89, 95 % CI: 0.68–1.18, *p* = 0.43), Lonsurf (*n* = 115, 21.9 %)(HR 0.87, 95 % CI: 0.65–1.18, *p* = 0.37), and BRAF inhibition (*n* = 12, 2.3 %)(HR 1.06, 95 % CI: 0.39–2.86, *p* = 0.91) were not associated with a survival benefit (Supplementary Figure 2).

### Molecular profiling of tumor tissue in patients with colorectal peritoneal metastases

Tumor mutational status was available for 442 (87.0 %) patients through clinical sequencing assays. At least 50 genes were tested in 418 (82.2 %) patients, and 313 (61.6 %) had over 100 genes tested (Supplementary Table 3). Plasma ctDNA testing was available for 145 patients, collected a median of 262 (IQR: 37–773) days from the diagnosis of CPM.

**Fig. 2:**
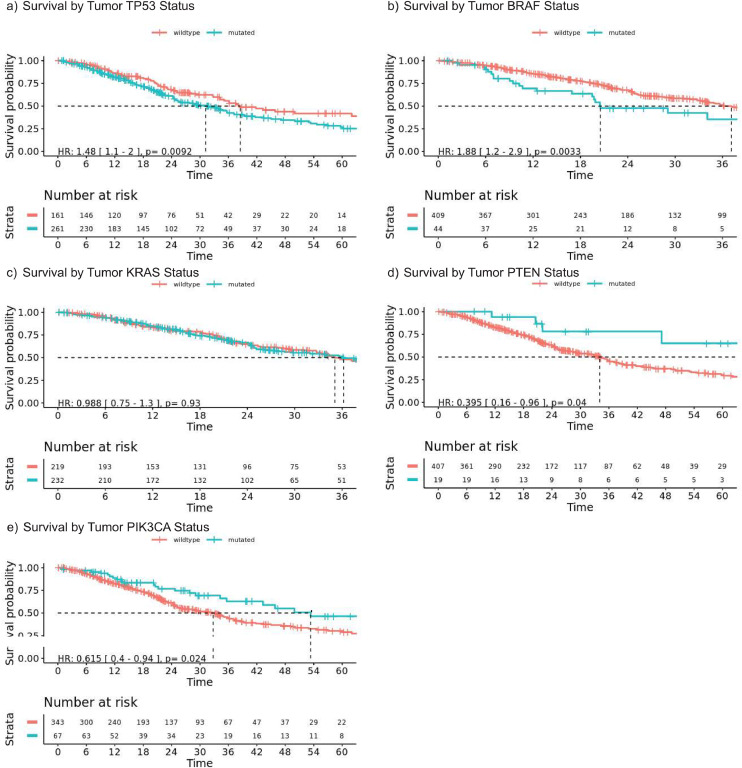
Associations between survival after peritoneal metastasis diagnosis and tumor tissue mutation status. (a) *TP53* wild-type patients had a longer median OS than mutated (38.5 versus 31.2 months, *p* = 0.0092). (b) Patients with mutated BRAF tumors had a shorter median overall survival (OS) (20.5 months versus 37.1 months, *p* = 0.0033). (c) KRAS mutation status was not associated with a difference in OS (mutated: 35.1 months versus wildtype 36.2 months, *p* = 0.93). (d) Patients with *PTEN* mutations had a longer median OS than wild-type patients (108.2 versus 34.1 months, *p* = 0.040). (e) Patients with *PIK3CA* mutations had a longer median OS than wild-type patients (53.4 versus 34.0 months, *p* = 0.024).

*TP53* (61.8 %) was the most commonly mutated gene identified in tumor tissue and was associated with worse OS after adjustment for other risk factors (Supplementary Table 2, Fig. 2a). Of note, *APC* was only covered in one of the tissue sequencing panels, and so its mutation frequency is not known in this total cohort. Mutations in *BRAF* were identified in 9.7 % of patients, 88.9 % of which were p.V600E, and were associated with worse OS (Fig. 2b). *KRAS* mutations were identified in 51.4 % of patients: 22.5 % p.G12D, 22.3 % p.G12V, 17.6 % p.G13D, 7.9 % p.Q61H, 6.2 % p.G12C, and 23.5 % other; and were not associated with OS (Fig. 2c). Similarly, there was no difference in overall survival based on the specific *KRAS* mutation classification as described by Tonello et al. (MUT1:G12R, G13A, G13C, G13V, Q61H, K117N, A146V; MUT2 (G12A, G12C, G12D, G12S, G12V, G13D, A59E, A59V, A146T) (Supplementary Figure 3) [11]. *KRAS* and *TP53* co-mutation occurred in 133 patients and were not associated with OS compared to mutation in one of the two genes (HR 1.28, 95 %CI: 0.94–1.74, *p* = 0.12). *PTEN* mutations known or inferred to be inactivating were identified in 4.5 % of patients and associated with longer OS (Fig. 2d) [16,17]. After adjustment for other mutations associated with OS on univariate analysis to control for biologically significant co-mutation or exclusive mutations, PTEN inactivation remained associated with improved OS (HR 0.31, 95 % CI: 0.13–0.77, *p* = 0.01, Supplementary Table 2). *PIK3CA* mutations associated with gain of function were identified in 16.2 % of patients and were associated with better OS on univariable analysis (Fig. 2e), but not after adjustment for other risk factors (Supplementary Table 2).

Additionally, we studied mutational profiles of these patients by histology to identify mutational profiles specific to mucinous (*n* = 161) and SRC (*n* = 34) histologic subtypes. These mutational profiles are described in Supplementary Tables 6 and 7. There were no statistically significant associations between survival and specific mutations in SRC. There was, however, significantly diminished survival associated with mucinous tumors harboring either *BRAF* mutations (HR 2.56, 95 %CI: 1.26–5.20, *p* = 0.01) or *TP53* mutations (HR 1.65, 95 %CI: 1.04–2.62, *p* = 0.03).

### Mutations and survival with colorectal liver metastases

**Fig. 3:**
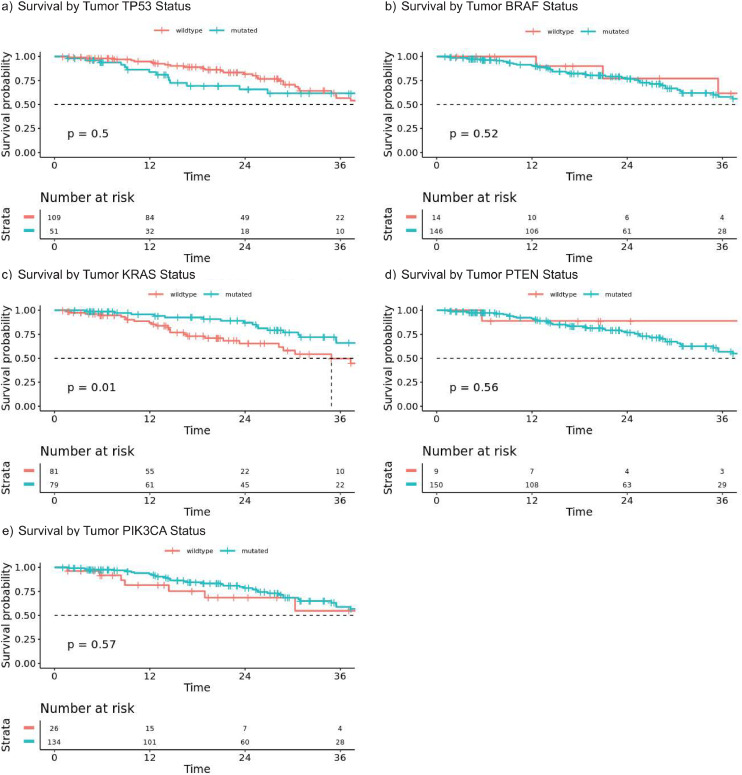
Overall survival of patients with unresected colorectal liver metastases and no peritoneal metastases. (a) *TP53* wild-type patients had similar median OS to mutated (50.0 months versus 41.2 months, *p* = 0.50). (b) Patients with mutated BRAF tumors had a similar median overall survival (OS) (Not reach versus 44.6 months, *p* = 0.522). (c) KRAS mutation status was associated with a shorter median OS (34.9 months versus 46.0 months, *p* = 0.012). (d) *PTEN* mutations was not associated with a difference in OS (44.6 months versus not reached, *p* = 0.56). (e) Patients with PIK3CA mutations had a similar median OS to wild-type patients (44.6 months versus not reached, *p* = 0.57).

Median age at diagnosis of metastasis, gender, ethnicity and race did not differ between patients with CPM or the 160 patients with CLM alone. Median OS for the CLM cohort was comparable to OS for CPM patients with no additional sites of metastasis (HR 1.08, 95 % CI 0.67–1.29, *p* = 0.66). Patients with CLM alone were less likely to have mucinous (10.8 % vs 36.5 %) or signet ring cell carcinomas (0.6 % vs 9.0 %) compared to patients with CPM alone (*p* < 0.001). CLM patients were less likely to have poorly differentiated tumors (8.3 % vs 19.4 %, *p* = 0.001). Amongst the CLM cohort, *KRAS* mutation was associated with significantly reduced survival in the CLM cohort as compared to the CPM group (34.8 vs. 46.0 months, HR 0.48 [0.3–0.9], *p* = 0.01). While *TP53* and *BRAF* were not associated with reduced OS in this cohort of patients. Survival curves stratified by mutation status are shown in Fig. 3.

### Circulating tumor DNA profiles associated with colorectal peritoneal metastases

**Fig. 4:**
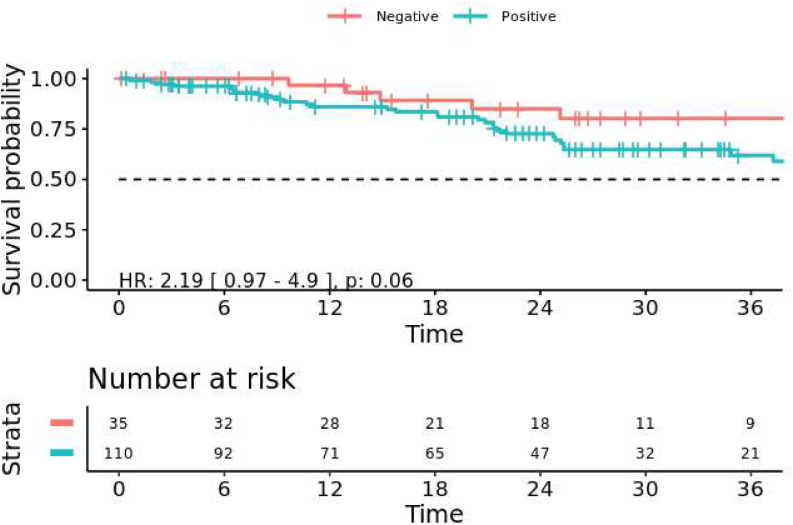
Overall survival after diagnosis of carcinomatosis by ctDNA status in patients with peritoneal disease. Patients with ctDNA detected had a trend towards worse survival (HR 2.19, 95 % CI: 0.97–4.94, *p* = 0.060).

Plasma cfDNA testing was performed on 145 patients from the overall CPM cohort of 526 patients. One hundred and ten (75.9 %) of these patients had a detectable mutation. Average time from diagnosis of peritoneal metastasis to ctDNA sequencing was 4.7 months. Timing of first test after diagnosis did not differ between patients with detectable mutations and those without mutations identified (Table 2). Liver metastases were associated with increased ctDNA detection rates, whereas mucinous histology was associated with decreased rates (Table 2). Of the 52 patients with CPM and CLM, 46 (88 %) had detectable ctDNA, compared to 62 (66 %) of the 94 patients with CPM alone (*p* < 0.0001). Similar to the tissue profiling, *TP53* was the most commonly mutated gene (*n* = 80, 52.6 %), followed by *APC* (*n* = 68, 44.7 %), and *KRAS* (*n* = 46, 30 %). Detection of any mutated ctDNA was associated with a trend towards worse survival (Fig. 4, *p* = 0.06). The maximum variant allele frequency within individual patients’ ctDNA was not associated with their OS (HR 0.99 per % VAF, 95 % CI: 0.97–1.01, *p* = 0.17) (Supplementary Figure 4)\

**Table 2:** Patient characteristics associated with somatic mutation detection in ctDNA.

	ctDNA+(*n* = 110)	ctDNA- (*n* = 35)	*p*
Age at CPM Diagnosis, years, median (IQR)	54.2 (46.1–64.0)	52.0 (38.0–61.5)	0.18
Months from Diagnosis to First ctDNA test	4.6 (0.9–20.4)	5.1 (1.0–20.1)	0.95
Gender, n ( %)			0.87
Male	67 (79)	18 (21)	0.43
Female	43 (72)	17 (28)	
Race, n ( %)			0.28
White/Caucasian	70 (74)	25 (26)	
Black/African	19 (90)	2 (10)	
Asian	6 (29)	15 (71)	
American Native	1 (100)	0 (0.0)	
Other/Unknown	14 (82)	3 (18)	
Ethnicity, n ( %)			0.86
Hispanic/Latino	17 (81)	4 (19)	
Other	88 (75)	30 (25)	
Unknown	1 (100)	0 (0.0)	
Tumor Grade, n ( %)			0.86
Well-Differentiated	1 (100)	0 (0.0)	
Moderately Diff.	82 (79)	22 (21)	
Poorly Diff.	17 (74)	6 (26)	
Unknown	10 (83)	2 (17)	
Histology, n ( %)			0.002
Mucinous	20 (59)	14 (41)	
Signet Ring Cell	8 (57)	6 (43)	
Adenocarcinoma NOS	82 (85)	15 (15)	
Other Metastases, n ( %)			
None			
Liver	46 (88)	6 (12)	0.014
Lung	14 (93)	1 (7)	0.18
Smoking History			0.85
Yes	40 (74)	14 (26)	
No	70 (77)	21 (23)

## Discussion

Here we describe the genomic characterization of a cohort of patients with unresectable peritoneal metastases of colorectal adenocarcinoma origin treated with contemporary multiagent chemotherapy regimens. Notably, this group displayed a median OS of 37.5 months, which is considerably longer than historic reports that describe survival of 13.5 to 18.8 months, and similar to our cohort of T4 primary colon patients who developed peritoneal metastases [18,19]. While this effect is multifactorial, earlier detection of primary or recurrent metastatic disease, the increased utilization of multidisciplinary care, and multi-drug chemotherapy are likely strong contributing factors. The majority of patients in this series received multi-drug regimens combining 5-Fluorouracil with oxaliplatin, irinotecan, anti-*EGFR* antibodies or anti-VEGF antibodies. Intriguingly patients who received FOLFIRI and/or Bevacizumab demonstrated improved survival to those not receiving these agents. Although this may represent selection bias to some extent as these agents are typically given at later lines of therapy it does suggest important efficacy of these agents in peritoneal metastases. In addition, improved surveillance with multi-detector CT has enhanced the ability to detect peritoneal disease at the earliest stages of development, potentially improving survival by controlling disease burden and forestalling the development of life-limiting complications such as malignant bowel obstruction [20]. Whether this improved sensitivity results in lead time bias or true improvements in disease management over time remains to be determined. However, this reported OS is similar to unresectable metastatic CRC treated with modern multi-drug regimens and brings into question the classic teaching that peritoneal metastases are associated with a worse prognosis than other sites of metastatic disease [21,22]. Aside from quantifying improvements in survival for these patients under contemporary standard of care, this cohort also allowed us to evaluate the prognostic value of tumoral and circulating tumor DNA mutations.

Intriguingly, mutational status allows for prognostication of CPM, with notable differences compared to other sites of metastases such as CLM. Whereas KRAS mutations and APC wild-type status are associated with worse outcomes for CLM, in this cohort of CPM we did detect differences in survival with this mutational status. Although notably there have been previously published associations with KRAS mutational status being associated with diminished survival in patients with peritoneal metastases. Although notably there have been previously published associations with *KRAS* mutational status being associated with diminished survival in patients with peritoneal metastases [[23], [24], [25]]. Additionally, location of KRAS point mutation, which has been linked to prognosis in other series, [11,26] did not impact survival in this cohort. Conversely, *BRAF* and *TP53* mutations were associated with worse OS, as has been demonstrated in other historic cohorts of metastatic CRC [23,[27], [28], [29]], *PIK3CA* gain-of-function and *PTEN* loss-of-function tumor mutations were associated with improved outcomes, suggesting potential targeted therapy options for CRCs that develop peritoneal dissemination. *GNAS* mutation, which is reported in <1 % of advanced CRC, was noted in 6 % of our cohort [30].

A relatively high proportion of KRAS mutant patients in our series harbored G12C substitutions. The KRAS G12C inhibitor adagrasib has shown promise in recently published trials with objective response rates as high as 46 % when combined with the EGFR inhibitor cetuximab [31,32]. The addition of KRAS-targeted therapy to the CRC armamentarium may further improve survival for patients with CPM, which already approached that of patients with CLM in this highly selected cohort. These data also call into question the use of CPM as a common exclusion criteria in clinical trials [33]. While more difficult to study from a target lesion perspective, a subset of patients with CPM may benefit significantly from KRAS inhibitors and other therapies under investigation.

Although a trend toward ctDNA positivity being negatively associated with survival, the maximum detected VAF were not associated with OS in this series. Interestingly, detection of circulating ctDNA *KRAS* mutations were associated with abbreviated survival. The emergence of *KRAS* mutation in ctDNA has been previously shown to correspond to poor outcomes, presumably due to anti-*EGFR* resistance [34]. While the biology of CPM is distinct from that of CLM, these findings demonstrate that mutations detected in ctDNA can also be used as a prognostic indicator in these patients. However, it should be noted that the sensitivity of ctDNA may be lower in patients with disease limited to the peritoneum, as evidenced by a 66 % detection rate in patients with CPM in this study. Furthermore, the actionability of ctDNA-based mutation profiles has not yet been demonstrated in prospective trials. At this juncture, the limitations of ctDNA in CPM have led us to utilize this new technology with caution.

Although these patients represent a cohort with unresectable metastatic disease, future work may inform decision making behind cytoreductive surgery, similar to their utility in surgical planning of the resection of colorectal liver metastases. Despite the improved survival in this cohort, it remains shorter than the 41-month median OS reported in the PRODIGE-7 trial of cytoreduction compared to cytoreduction with hyperthermic intraperitoneal oxaliplatin chemotherapy [3]. An improved patient understanding of tumor biology will likely be able to further expand the benefit of cytoreduction as patient selection and systemic disease control continue to improve. This is, of course, only possible with current efforts ongoing to define the biology of these tumors by our group and others [[35], [36], [37]]. Tumor burden (peritoneal cancer index) was not assessed in these patients, given that they were deemed unresectable and not surgically explored. This may be a confounding factor when comparing survival of patients who were suitable for complete cytoreduction. While these findings do not change the decision making behind consideration for cytoreduction, it does speak to the need to continually weigh the morbidity of cytoreduction against its benefit as compared to systemic chemotherapy.

### Limitations

This work must be considered in the context of its limitations. This is a retrospective analysis of patients treated at a single institution and thus reflects the treatment trends and decisions of the multi-disciplinary tumor board at the MD Anderson Cancer Center. Selection bias towards patients with improved performance status and lower disease burden sufficient to undergo systemic therapy and also be referred to a specialized cancer center may lead to longer OS in this cohort than the overall population of patients with peritoneal disease. This work also utilized the unique and previously validated Foundry platform that allows for robust review of medical records without the error introduced by hand review. It does, however, introduce limitations on the ability to hand review subsets of patients. However, a study of primary T4 colon cancer patients treated at our institution identified a median OS after diagnosis with carcinomatosis of 28.9 (IQR 15.3–41.4) months, suggesting the improved OS found in the current study is not solely due to referral bias [19]. Progression free survival is incompletely recorded given our institution's role as a referral center. We therefore elected to report overall survival as the majority of deaths would be secondary to malignancies in this group and was reliably recorded by the tumor registry using clinical and non-clinical external sources.

The cohort spans multiple distinct tumor tissue profiling assays, including different methodologies and variable coverage, especially in less common mutations. Moreover, this coverage increased over time potentially introducing lead time bias to these less common mutations only included in extended panels. Only a minority of tissue sequencing was performed on PM tissue. The assays were not validated for the detection of copy number losses. All assays were DNA-based and included no-to-limited coverage of intronic breakpoints for fusion detection.

### Conclusions

Moving forward, continued study of colorectal peritoneal metastases is critical to improve survival for this challenging subset of patients. Unfortunately, these patients have been excluded from clinical trials with some regularity [33]. It will be important to include these patients in trials of systemic therapy and cytoreduction.

**Supplementary Figure 1. Flowchart showing selection of the peritoneal patient cohort.** Of 508 patients with colorectal peritoneal metastases, 508 were managed non-operatively and included in this study's analysis cohort.

**Supplementary Figure 2. Overall survival associations with type of chemotherapy receipt within the peritoneal metastasis cohort.** (a) Patients receiving FOLFIRI during their treatment course compared to those not receiving FOLFIRI (HR 0.69, 95 % CI: 0.52–0.90, *p* = 0.008). (b) Patients receiving Bevacizumab during their treatment course compared to those not receiving Bevacizumab (HR 0.73, 95 % CI 0.56–0.96, *p* = 0.023). (c) Patients receiving FOLFOX during their treatment course compared to those not receiving FOLFOX l (HR 0.81, 95 % CI: 1.23–0.60, *p* = 0.17). (d) Patients receiving anti-EGFR therapy during their treatment course compared to those not receiving anti-EGFR therapy (*n* = 148, 28.1 %)(HR 0.89, 95 % CI: 0.68–1.18, *p* = 0.43). (e) Patients receiving Lonsurf during their treatment course compared to those not receiving Lonsurf (*n* = 115, 21.9 %)(HR 0.87, 95 % CI: 0.65–1.18, *p* = 0.37). (f) Patients receiving BRAF inhibition during their treatment course compared to those not receiving BRAF inhibition (*n* = 12, 2.3 %)(HR 1.06, 95 % CI 0.39–2.86, *p* = 0.91). Number of patients does not add to total cohort (*n* = 526) when retrospective review of regimen was unclear or unavailable.

**Supplementary Figure 3: Overall survival stratified by mutational subtype of KRAS mutation within the peritoneal metastasis cohort.** There was no difference in OS from time of peritoneal metastasis diagnosis based on subtype of KRAS mutational subtypes (MUT1:G12R, G13A, G13C, G13V, Q61H, K117N, A146V; MUT2 (G12A, G12C, G12D, G12S, G12V, G13D, A59E, A59V, A146T) (HR 1.03 [0.79–1.4], *p* = 0.81).

**Supplementary Figure 4. Variant allele frequency in circulating cell-free DNA within the peritoneal metastasis cohort.** Histogram of by maximum variant allele frequency of screened mutations identified in blood samples. List of mutations screened are provided in Supplemental Table 1.

**Supplementary Figure 1. Flowchart showing selection of the peritoneal patient cohort.** Of 508 patients with colorectal peritoneal metastases, 508 were managed non-operatively and included in this study's analysis cohort.

**Supplementary Figure 2. Overall survival associations with type of chemotherapy receipt within the peritoneal metastasis cohort.** (a) Patients receiving FOLFIRI during their treatment course compared to those not receiving FOLFIRI (HR 0.69, 95 % CI: 0.52–0.90, *p* = 0.008). (b) Patients receiving Bevacizumab during their treatment course compared to those not receiving Bevacizumab (HR 0.73, 95 % CI 0.56–0.96, *p* = 0.023). (c) Patients receiving FOLFOX during their treatment course compared to those not receiving FOLFOX l (HR 0.81, 95 % CI: 1.23–0.60, *p* = 0.17). (d) Patients receiving anti-EGFR therapy during their treatment course compared to those not receiving anti-EGFR therapy (*n* = 148, 28.1 %)(HR 0.89, 95 % CI: 0.68–1.18, *p* = 0.43). (e) Patients receiving Lonsurf during their treatment course compared to those not receiving Lonsurf (*n* = 115, 21.9 %)(HR 0.87, 95 % CI: 0.65–1.18, *p* = 0.37). (f) Patients receiving BRAF inhibition during their treatment course compared to those not receiving BRAF inhibition (*n* = 12, 2.3 %)(HR 1.06, 95 % CI 0.39–2.86, *p* = 0.91). Number of patients does not add to total cohort (*n* = 526) when retrospective review of regimen was unclear or unavailable.

**Supplementary Figure 3: Overall survival stratified by mutational subtype of KRAS mutation within the peritoneal metastasis cohort.** There was no difference in OS from time of peritoneal metastasis diagnosis based on subtype of KRAS mutational subtypes (MUT1:G12R, G13A, G13C, G13V, Q61H, K117N, A146V; MUT2 (G12A, G12C, G12D, G12S, G12V, G13D, A59E, A59V, A146T) (HR 1.03 [0.79–1.4], *p* = 0.81).

**Supplementary Figure 4. Variant allele frequency in circulating cell-free DNA within the peritoneal metastasis cohort.** Histogram of by maximum variant allele frequency of screened mutations identified in blood samples. List of mutations screened are provided in Supplemental Table 1.

## CRediT authorship contribution statement

**Michael G. White:** Writing – review & editing, Writing – original draft, Visualization, Methodology, Investigation, Conceptualization. **Reed I. Ayabe:** Writing – review & editing, Writing – original draft, Methodology, Formal analysis. **Mohammad A. Zeineddine:** Writing – review & editing, Investigation, Data curation. **Fadl A. Zeineddine:** Writing – review & editing, Data curation. **Abdelrahman M.G. Yousef:** Writing – review & editing, Data curation. **Mahmoud Yousef:** Writing – review & editing, Data curation. **Norman J. Galbraith:** Writing – review & editing. **J․Bryan Iorgulescu:** Writing – review & editing, Methodology. **Christopher Scally:** Writing – review & editing, Conceptualization. **Keith Fournier:** Writing – review & editing, Supervision, Investigation, Conceptualization. **Timothy E. Newhook:** Writing – review & editing, Conceptualization. **Nancy Y. You:** Writing – review & editing, Conceptualization. **Jason Willis:** Writing – review & editing. **Scott Kopetz:** Writing – review & editing, Supervision, Conceptualization. **George J. Chang:** Writing – review & editing, Supervision. **John Paul Shen:** Writing – review & editing, Investigation, Formal analysis, Conceptualization. **Abhineet Uppal:** Writing – review & editing, Writing – original draft, Visualization, Software, Formal analysis, Data curation, Conceptualization.

## Declaration of competing interest

The authors declare that they have no known competing financial interests or personal relationships that could have appeared to influence the work reported in this paper.

## Appendix Supplementary materials

Image, application 1

Image, application 2

Image, application 3

Image, application 4

Image, application 5

Image, application 6

Image, application 7
