# Supplementary material for: Tumoral and circulating genomic landscape inform survival differences in colorectal carcinomatosis
Source: Transl Oncol. 2025 Apr 3;55:102379. doi: 10.1016/j.tranon.2025.102379 (PMC12002894; doi:10.1016/j.tranon.2025.102379)
Supplement: Supplementary file 1 [file mmc1.docx]

| **Supplementary Table 1. Multivariable cox regression of the clinicopathologic factors associated with overall survival among patients with CPM** | | |
| --- | --- | --- |
| **Factor** | **HR (95% CI)** | **p value** |
| Age at diagnosis, years | 1.02/year (1.01-1.04) | 0.004 |
| Liver metastasis | 1.67 (1.26-2.22) | <0.001 |
| Male sex | 1.37 (1.05-1.78) | 0.019 |
| Signet ring cell histology | 2.35 (1.41-3.92) | 0.001 |
| Poorly differentiated | 2.08 (1.42-3.04) | <0.001 |

| **Supplementary Table 2. Association of overall survival with mutations in the 10 most frequently altered genes in tumor tissue of patients with CPM** | | | | | |
| --- | --- | --- | --- | --- | --- |
| **Gene** | **n (%) with mutation** | **Unadjusted HR (95% CI)** | **Corrected *p*** | **Adjusted* HR (95% CI)** |  |
| *TP53* | 261 (61.8) | 1.51 (1.12-2.02) | 0.005 | 1.66 (1.21-2.27) | 0.001 |
| *PIK3CA* | 67 (16.2) | 0.65 (0.43-0.98) | 0.038 | 0.66 (0.43-1.00) | 0.053 |
| *BRAF* | 44 (9.7) | 1.88 (1.23-2.86) | 0.003 | 1.79 (1.17-2.74) | 0.007 |
| *PTEN* | 19 (4.5) | 0.41 (0.17-0.99) | 0.049 | 0.31 (0.13-0.77) | 0.011 |
|  |  |  |  |  |  |
| *APC* | 125 (52.3) | 0.78 (0.56-1.08) | 0.778 |  |  |
| *KRAS* | 227 (51.4) | 0.94 (0.72-1.24) | 0.676 |  |  |
| *SMAD4* | 68 (16.0) | 0.78 (0.53-1.16) | 0.214 |  |  |
| *FBXW7* | 37 (8.7) | 1.23 (0.76-1.99) | 0.410 |  |  |
| *ATM* | 31 (7.3) | 0.95 (0.56-1.61) | 0.856 |  |  |
| *GNAS* | 26 (6.1) | 1.22 (0.72-2.08) | 0.455 |  |  |
| *Adjusted for age at CPM diagnosis, non-peritoneal metastasis, gender, histologic subtype, and grade. Note: Correction for multiple testing was used to determine *p* values. Some genes are not covered in all assays used during the study period. | | | | | |

| **Supplementary Table 3. Genes analyzed in solid tumor specimens and frequency of mutation with CPM** | | | |
| --- | --- | --- | --- |
| **Gene** | **Not detected, n** | **Somatic Mutation detected, n** | **%** |
| *TP53* | 161 | 261 | 61.85 |
| *APC* | 107 | 123 | 53.48 |
| *KRAS* | 210 | 227 | 51.95 |
| *PIK3CA* | 343 | 67 | 16.34 |
| *SMAD4* | 355 | 68 | 16.08 |
| *BRAF* | 395 | 44 | 10.02 |
| *FBXW7* | 389 | 37 | 8.69 |
| *RNF43* | 178 | 16 | 8.25 |
| *ATM* | 376 | 31 | 7.62 |
| *SMARCA4* | 182 | 12 | 6.19 |
| *GNAS* | 402 | 26 | 6.07 |
| *SETD2* | 183 | 11 | 5.67 |
| *NOTCH3* | 184 | 10 | 5.15 |
| *SLX4* | 184 | 10 | 5.15 |
| *BRCA2* | 299 | 15 | 4.78 |
| *POLE* | 185 | 9 | 4.64 |
| *CREBBP* | 186 | 9 | 4.62 |
| *PTEN* | 407 | 19 | 4.46 |
| *PIK3R1* | 302 | 12 | 3.82 |
| *ARID1A* | 187 | 7 | 3.61 |
| *TSC2* | 303 | 11 | 3.50 |
| *NOTCH1* | 404 | 13 | 3.12 |
| *FANCA* | 188 | 6 | 3.09 |
| *AKT3* | 188 | 6 | 3.09 |
| *BRCA1* | 305 | 9 | 2.87 |
| *BAP1* | 305 | 9 | 2.87 |
| *CTNNB1* | 414 | 11 | 2.59 |
| *ATR* | 189 | 5 | 2.58 |
| *FANCI* | 189 | 5 | 2.58 |
| *PTCH1* | 306 | 8 | 2.55 |
| *NF1* | 307 | 8 | 2.54 |
| *ERBB2* | 416 | 9 | 2.12 |
| *NRAS* | 425 | 9 | 2.07 |
| *ATRX* | 190 | 4 | 2.06 |
| *CDK12* | 190 | 4 | 2.06 |
| *MSH6* | 190 | 4 | 2.06 |
| *NOTCH2* | 190 | 4 | 2.06 |
| *FANCD2* | 190 | 4 | 2.06 |
| *NTRK3* | 198 | 4 | 1.98 |
| *MSH2* | 308 | 6 | 1.91 |
| *RB1* | 414 | 7 | 1.66 |
| *TET2* | 119 | 2 | 1.65 |
| *MLH1* | 418 | 7 | 1.65 |
| *WT1* | 120 | 2 | 1.64 |
| *TSC1* | 309 | 5 | 1.59 |
| *CDKN2B* | 190 | 3 | 1.55 |
| *RAD50* | 191 | 3 | 1.55 |
| *MTOR* | 310 | 4 | 1.27 |
| *AR* | 310 | 4 | 1.27 |
| *ESR1* | 310 | 4 | 1.27 |
| *NF2* | 310 | 4 | 1.27 |
| *KIT* | 398 | 5 | 1.24 |
| *ALK* | 420 | 5 | 1.18 |
| *MRE11A* | 174 | 2 | 1.14 |
| *FGF3* | 191 | 2 | 1.04 |
| *NBN* | 192 | 2 | 1.03 |
| *FGF19* | 192 | 2 | 1.03 |
| *RAD51B* | 192 | 2 | 1.03 |
| *NTRK2* | 200 | 2 | 0.99 |
| *PPP2R1A* | 311 | 3 | 0.96 |
| *RAF1* | 311 | 3 | 0.96 |
| *STK11* | 415 | 4 | 0.95 |
| *RET* | 416 | 4 | 0.95 |
| *MAP2K1* | 312 | 3 | 0.95 |
| *U2AF1* | 312 | 3 | 0.95 |
| *DCUN1D1* | 113 | 1 | 0.88 |
| *CDH1* | 232 | 2 | 0.85 |
| *MYO18A* | 119 | 1 | 0.83 |
| *ZNF217* | 119 | 1 | 0.83 |
| *SOX2* | 119 | 1 | 0.83 |
| *IL6* | 119 | 1 | 0.83 |
| *RPS6KB1* | 119 | 1 | 0.83 |
| *CDKN2A* | 414 | 3 | 0.72 |
| *MET* | 415 | 3 | 0.72 |
| *EGFR* | 421 | 3 | 0.71 |
| *ERBB4* | 422 | 3 | 0.71 |
| *PDGFRA* | 422 | 3 | 0.71 |
| *KDR* | 422 | 3 | 0.71 |
| *AKT1* | 423 | 3 | 0.70 |
| *FLT3* | 423 | 3 | 0.70 |
| *DDR2* | 312 | 2 | 0.64 |
| *MYCN* | 312 | 2 | 0.64 |
| *MAX* | 312 | 2 | 0.64 |
| *MYC* | 312 | 2 | 0.64 |
| *RHOA* | 312 | 2 | 0.64 |
| *PPARG* | 312 | 2 | 0.64 |
| *SF3B1* | 313 | 2 | 0.63 |
| *PDGFRB* | 193 | 1 | 0.52 |
| *MAP2K4* | 193 | 1 | 0.52 |
| *RICTOR* | 193 | 1 | 0.52 |
| *AXL* | 193 | 1 | 0.52 |
| *PMS2* | 193 | 1 | 0.52 |
| *CDKN1B* | 193 | 1 | 0.52 |
| *CHEK1* | 193 | 1 | 0.52 |
| *CCND3* | 193 | 1 | 0.52 |
| *NTRK1* | 201 | 1 | 0.50 |
| *FGFR3* | 409 | 2 | 0.49 |
| *SMO* | 421 | 2 | 0.47 |
| *SMARCB1* | 421 | 2 | 0.47 |
| *FGFR1* | 422 | 2 | 0.47 |
| *IDH1* | 426 | 2 | 0.47 |
| *VHL* | 229 | 1 | 0.43 |
| *ABL1* | 232 | 1 | 0.43 |
| *MDM4* | 310 | 1 | 0.32 |
| *GATA2* | 310 | 1 | 0.32 |
| *BTK* | 312 | 1 | 0.32 |
| *NFE2L2* | 312 | 1 | 0.32 |
| *RHEB* | 312 | 1 | 0.32 |
| *CCND1* | 312 | 1 | 0.32 |
| *ERBB3* | 313 | 1 | 0.32 |
| *CCNE1* | 313 | 1 | 0.32 |
| *MDM2* | 313 | 1 | 0.32 |
| *EZH2* | 419 | 1 | 0.24 |
| *IDH2* | 419 | 1 | 0.24 |
| *CSF1R* | 424 | 1 | 0.24 |
| *FGFR2* | 424 | 1 | 0.24 |
| *PTPN11* | 424 | 1 | 0.24 |
| *JAK3* | 419 | 0 | 0.00 |
| *HNF1A* | 424 | 0 | 0.00 |
| *MPL* | 230 | 0 | 0.00 |
| *PAX5* | 121 | 0 | 0.00 |

| **Supplementary Table 4. Genes analyzed in peripheral plasma specimens and frequency of mutation for patients with CPM** | | | |
| --- | --- | --- | --- |
| **Gene** | **Wild-Type, n** | **Mutation, n** | **% Mutation** |
| *TP53* | 72 | 80 | 52.6 |
| *APC* | 84 | 68 | 44.7 |
| *KRAS* | 106 | 46 | 30.3 |
| *PIK3CA* | 132 | 20 | 13.2 |
| *SMAD4* | 134 | 18 | 11.8 |
| *BRAF* | 139 | 13 | 8.6 |
| *EGFR* | 142 | 10 | 6.6 |
| *NF1* | 144 | 8 | 5.3 |
| *BRCA2* | 145 | 7 | 4.6 |
| *ERBB2* | 145 | 7 | 4.6 |
| *GNAS* | 145 | 7 | 4.6 |
| *BRCA1* | 146 | 6 | 3.9 |
| *ARID1A* | 147 | 5 | 3.3 |
| *FBXW7* | 147 | 5 | 3.3 |
| *NRAS* | 147 | 5 | 3.3 |
| *MET* | 148 | 4 | 2.6 |
| *NTRK1* | 148 | 4 | 2.6 |
| *RB1* | 149 | 3 | 2.0 |
| *AR* | 149 | 3 | 2.0 |
| *CCNE1* | 149 | 3 | 2.0 |
| *MYC* | 149 | 3 | 2.0 |
| *NOTCH1* | 149 | 3 | 2.0 |
| *PTEN* | 149 | 3 | 2.0 |
| *PTPN11* | 149 | 3 | 2.0 |
| *RAF1* | 150 | 2 | 1.3 |
| *STK11* | 150 | 2 | 1.3 |
| *ALK* | 150 | 2 | 1.3 |
| *MAP2K1* | 150 | 2 | 1.3 |
| *MAPK1* | 150 | 2 | 1.3 |
| *MTOR* | 150 | 2 | 1.3 |
| *TERT* | 151 | 1 | 0.7 |
| *TSC1* | 151 | 1 | 0.7 |
| *VHL* | 151 | 1 | 0.7 |
| *CDK6* | 151 | 1 | 0.7 |
| *CTNNB1* | 151 | 1 | 0.7 |
| *DDR2* | 151 | 1 | 0.7 |
| *FGFR1* | 151 | 1 | 0.7 |
| *GNA11* | 151 | 1 | 0.7 |
| *IDH1* | 151 | 1 | 0.7 |
| *MAPK3* | 151 | 1 | 0.7 |
| *MPL* | 151 | 1 | 0.7 |
| *NTRK3* | 151 | 1 | 0.7 |
| *RAD51* | 151 | 1 | 0.7 |

| **Supplementary Table 5. Frequency of mutations in patients with carcinomatosis and those with liver metastases** | | | | | | | |
| --- | --- | --- | --- | --- | --- | --- | --- |
|  | **Colorectal Peritoneal Metastasis Patients** | | | **Colorectal Liver Metastases Patients** | | |  |
|  | Mut |  | % Mut | Mut |  | % Mut | p value |
| ***PIK3CA*** |  |  |  |  |  |  | 0.945 |
| E545K | 16 |  | 3.2 | 7 |  | 4.5 |  |
| H1047R | 13 |  | 2.6 | 4 |  | 2.5 |  |
| E542K | 10 |  | 2.0 | 4 |  | 2.5 |  |
| R88Q | 7 |  | 1.4 | 2 |  | 1.3 |  |
| Q546R | 3 |  | 0.6 | 1 |  | 0.6 |  |
|  |  |  |  |  |  |  |  |
| ***BRAF*** |  |  |  |  |  |  | 0.266 |
| V600E | 45 |  | 8.8 | 11 |  | 7.0 |  |
| Others | 8 |  | 1.6 | 4 |  | 2.5 |  |
|  |  |  |  |  |  |  |  |
| ***KRAS*** |  |  |  |  |  |  | 0.692 |
| G12D | 69 |  | 13.6 | 19 |  | 12.1 |  |
| G12V | 64 |  | 12.6 | 19 |  | 12.1 |  |
| G13D | 45 |  | 8.9 | 13 |  | 8.3 |  |
| G12C | 20 |  | 3.9 | 8 |  | 5.1 |  |
| Q61H | 18 |  | 3.5 | 2 |  | 1.3 |  |
|  |  |  |  |  |  |  |  |
| ***SMAD4*** |  |  |  |  |  |  | 0.045 |
| R361H | 20 |  | 4.0 | 2 |  | 1.3 |  |
| R361C | 7 |  | 1.4 | 5 |  | 3.2 |  |
| A118V | 6 |  | 1.2 | 0 |  | 0.0 |  |
| P356L | 4 |  | 0.8 | 0 |  | 0.0 |  |
| E538* | 2 |  | 0.4 | 0 |  | 0.0 |  |
|  |  |  |  |  |  |  |  |
|  |  |  |  |  |  |  |  |
| ***TP53*** |  |  |  |  |  |  | 0.23 |
| R175H | 34 |  | 6.7 | 7 |  | 4.5 |  |
| R248Q | 21 |  | 4.2 | 4 |  | 2.5 |  |
| R273H | 16 |  | 3.2 | 7 |  | 4.5 |  |
| R282W | 14 |  | 2.8 | 7 |  | 4.5 |  |
| R248W | 14 |  | 2.8 | 4 |  | 2.5 |  |
| R213* | 11 |  | 2.2 | 4 |  | 2.5 |  |
| R342* | 10 |  | 2.0 | 0 |  | 0.0 |  |
|  |  |  |  |  |  |  |  |
| ***FBXW7*** |  |  |  |  |  |  | 0.48 |
| R465C | 6 |  | 1.2 | 3 |  | 1.9 |  |
| R505C | 6 |  | 1.2 | 0 |  | 0.0 |  |
| R465H | 4 |  | 0.8 | 1 |  | 0.6 |  |
| R479Q | 3 |  | 0.6 | 2 |  | 1.3 |  |
| R568* | 2 |  | 0.4 | 2 |  | 1.3 |  |
|  |  |  |  |  |  |  |  |
| ***NRAS*** |  |  |  |  |  |  | 0.41 |
| G12D | 3 |  | 0.6 | 1 |  | 0.6 |  |
| Q61R | 3 |  | 0.6 | 1 |  | 0.6 |  |
| Q61K | 2 |  | 0.4 | 3 |  | 1.9 |  |
| E63K | 2 |  | 0.4 | 0 |  | 0.0 |  |
| G12C | 1 |  | 0.2 | 1 |  | 0.6 |  |
|  |  |  |  |  |  |  |  |
| ***PTEN*** |  |  |  |  |  |  | 0.40 |
| R173C | 2 |  | 0.4 | 0 |  | 0.0 |  |
| K267Rfs*9 | 1 |  | 0.2 | 0 |  | 0.0 |  |
| 1027-1G>A | 1 |  | 0.2 | 0 |  | 0.0 |  |
| P96L | 1 |  | 0.2 | 0 |  | 0.0 |  |
| Q87* | 1 |  | 0.2 | 0 |  | 0.0 |  |
|  |  |  |  |  |  |  |  |
| ***ERBB2*** |  |  |  |  |  |  | 0.40 |
| R678Q | 2 |  | 0.4 | 0 |  | 0.0 |  |
| V777L | 1 |  | 0.2 | 0 |  | 0.0 |  |
| D769H | 1 |  | 0.2 | 0 |  | 0.0 |  |
| S834N | 1 |  | 0.2 | 0 |  | 0.0 |  |
| K1096N | 1 |  | 0.2 | 0 |  | 0.0 |  |
|  |  |  |  |  |  |  |  |
| ***GNAS*** |  |  |  |  |  |  | 0.01 |
| R201H | 26 |  | 5.1 | 0 |  | 0.0 |  |
| R201C | 7 |  | 1.4 | 1 |  | 0.6 |  |

Mut: mutated, WT: wild-type
