# Supplementary figures and images for: Tumoral and circulating genomic landscape inform survival differences in colorectal carcinomatosis
Source: Transl Oncol. 2025 Apr 3;55:102379. doi: 10.1016/j.tranon.2025.102379 (PMC12002894; doi:10.1016/j.tranon.2025.102379)

**Supplementary Figure 1. Flowchart showing selection of the peritoneal patient cohort.**

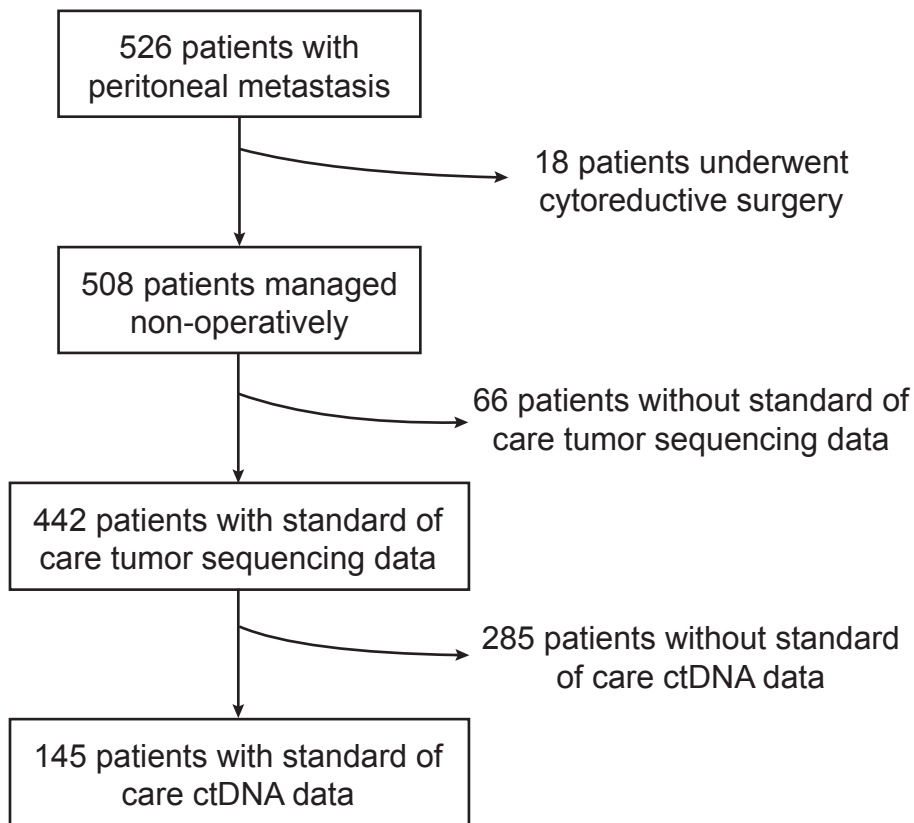

Supplement: Supplementary file 2 [file mmc2.pdf]
