# Supplementary material for: Tumoral and circulating genomic landscape inform survival differences in colorectal carcinomatosis
Source: Transl Oncol. 2025 Apr 3;55:102379. doi: 10.1016/j.tranon.2025.102379 (PMC12002894; doi:10.1016/j.tranon.2025.102379)

Supplementary Figure 2. Overall survival associations with type of chemotherapy receipt within the peritoneal metastasis cohort.

a. Survival by FOLFIRI receipt

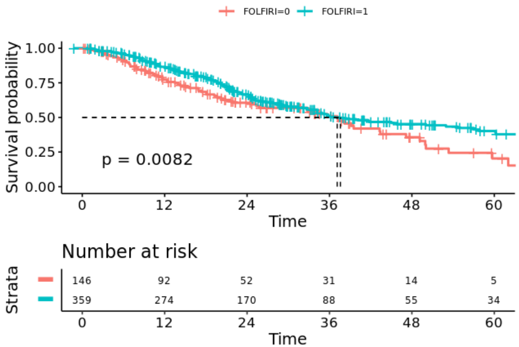

b. Survival by Bevacizumab receipt

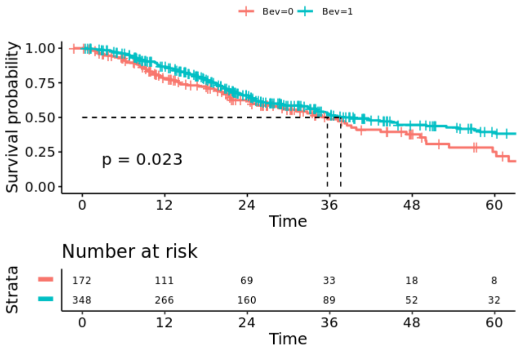

c. Survival by FOLFOX receipt

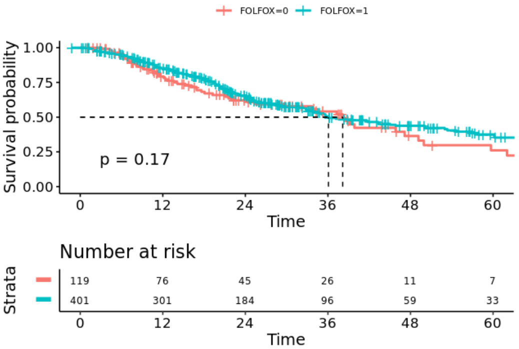

d. Survival by anti-EGFR receipt

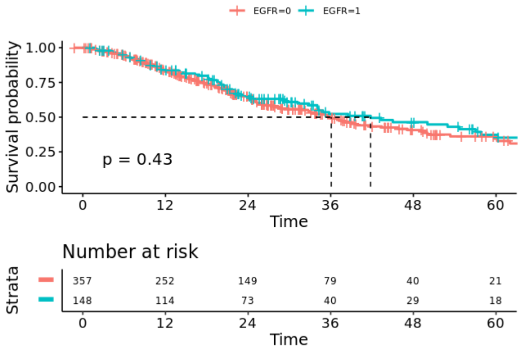

e. Survival by Lonsurf receipt

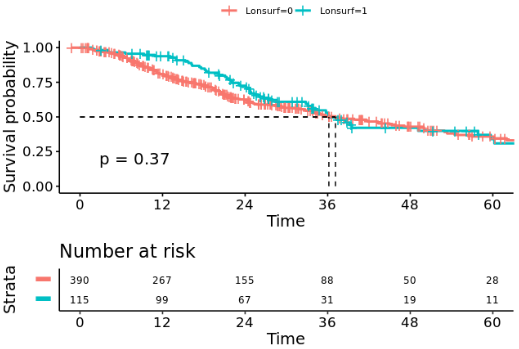

e. Survival by BRAF inhibition receipt

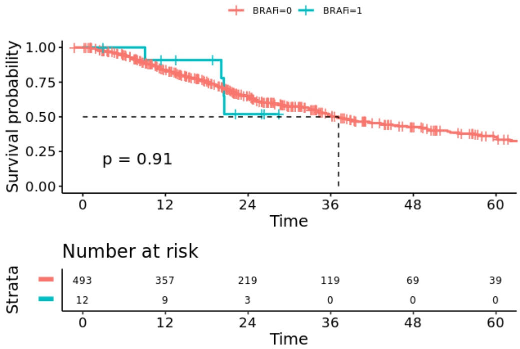

Supplement: Supplementary file 3 [file mmc3.pdf]
