# Supplementary material for: Tumoral and circulating genomic landscape inform survival differences in colorectal carcinomatosis
Source: Transl Oncol. 2025 Apr 3;55:102379. doi: 10.1016/j.tranon.2025.102379 (PMC12002894; doi:10.1016/j.tranon.2025.102379)

Supplementary Figure 3: Overall survival stratified by mutational subtype of KRAS mutation within the peritoneal metastasis cohort.

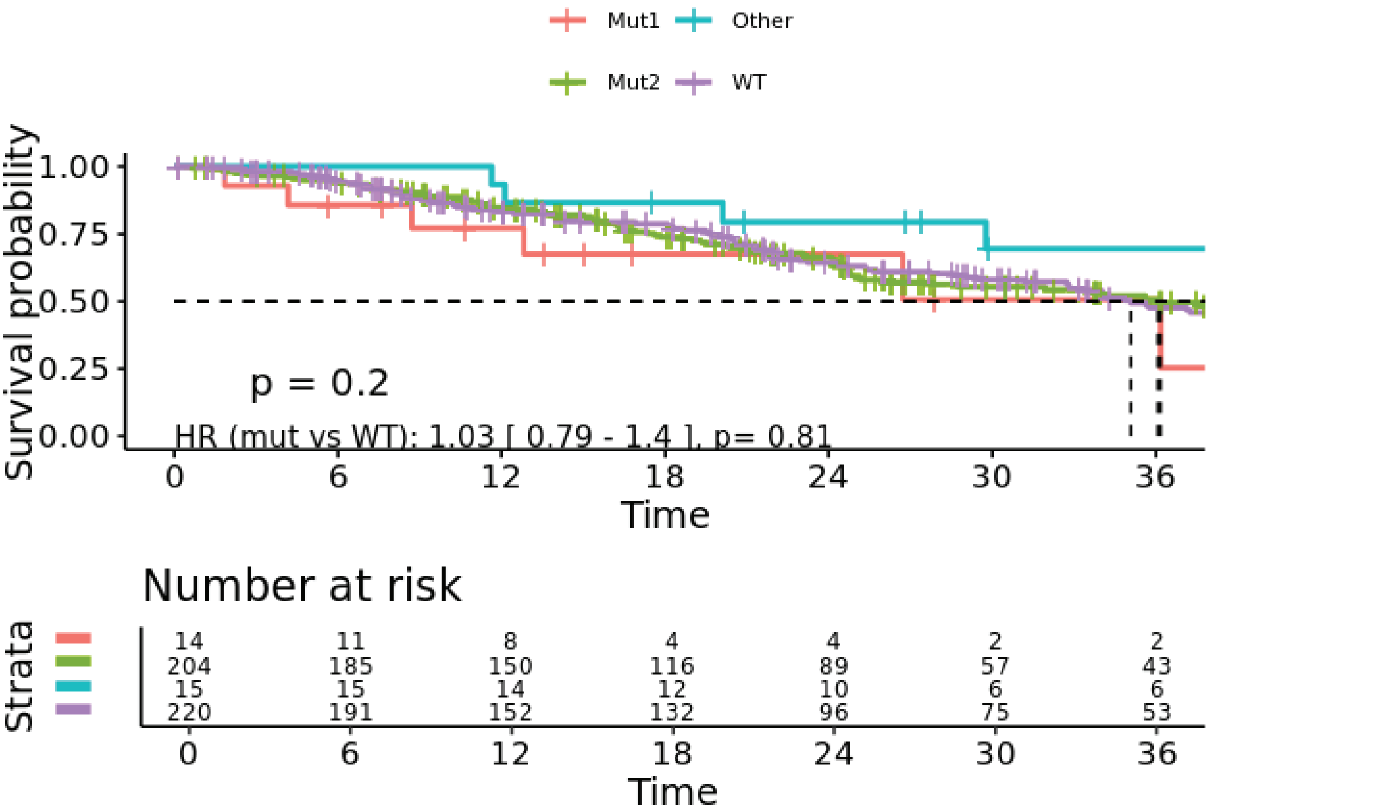

Supplement: Supplementary file 4 [file mmc4.pdf]
